# Adsorption Behaviors of Cationic Methylene Blue and Anionic Reactive Blue 19 Dyes onto Nano-Carbon Adsorbent Carbonized from Small Precursors

**DOI:** 10.3390/nano12111814

**Published:** 2022-05-25

**Authors:** Caizhen Liang, Qingshan Shi, Jin Feng, Junwei Yao, Hui Huang, Xiaobao Xie

**Affiliations:** Institute of Microbiology, Guangdong Academy of Sciences, State Key Laboratory of Applied Microbiology Southern China, Guangdong Provincial Key Laboratory of Microbial Culture Collection and Application, Guangdong Open Laboratory of Applied Microbiology, Guangzhou 510070, China; liangcz@gdim.cn (C.L.); shiqingshan@hotmail.com (Q.S.); fengj@gdim.cn (J.F.); yaojunwei@nwafu.edu.cn (J.Y.); huangh@gdim.cn (H.H.)

**Keywords:** nano-carbon adsorbent, small precursors, adsorption, methylene blue, reactive blue 19, selective capability

## Abstract

In this work, an innovative nano-carbon material (N-CM) adsorbent was reported for exploring its adsorption behaviors toward cationic methylene blue (MB) and anionic reactive blue 19 (RB19) pollutants. The proposed N-CM was synthesized by a one-step solvothermal treatment of citric acid and zinc gluconate small precursors. N-CM consists of nanosheets that have an advantageous specific surface area, large sp^2^/sp^3^ hybridized domains, and abundant nitrogen/oxygen-containing surface functional groups. The synergistic effects of these features are conducive to the MB and RB19 adsorption. Different from anionic RB19 adsorption (79.54 mg/g) by the cooperative π-π stacking and hydrogen bonding, cationic MB adsorbed onto N-CM mainly by the electrostatic attraction at the natural pH solution (> pH*_pzc_*), with an adsorption capacity up to 118.98 mg/g. Interestingly, both MB and RB19 adsorption conformed to the pseudo-second order kinetic (R^2^ ≥ 0.995) and Langmuir isothermal (R^2^ ≥ 0.990) models, accompanied by similar maximum monolayer adsorption capacities of 120.77 and 116.01 mg/g, respectively. Their adsorption processes exhibited spontaneously endothermic characteristics. Moreover, N-CM showed superior selective capability toward MB in different mixed dye systems, with high removal efficiencies of 73–89%. These results demonstrate that the high-performance carbon adsorbent prepared from small precursors via low-temperature carbonization shows great potentials in wastewater treatment.

## 1. Introduction

The rapid development of modern industrialization and economy advances people’s color perception, and thus accelerates the exploitation of various dyes with chemical inertness and fastness for satisfying different needs [1,2,3]. Methylene blue (MB), an aromatic heterocyclic compound, is a typical cationic dye that has been extensively applied in textile printing, dyeing, and cell staining [4]. However, it is also one of the main organic pollutants to water body and biologic chains [5]. In addition, reactive blue 19 (RB19), an anthraquinone compound with β-sulfatoethylsulfonyl group, is an important anionic reactive dye in the textile industry as well as a raw material for developing polymeric colorants, which is classified into the recalcitrant and toxic organic pollutants [6,7]. Their improper treatment and discharging from industrials are deteriorating the water body and threatening the ecological balance [7,8]. The dyes can enter the human body via the biologic chains due to their non-degradation, bioaccumulation and high water-solubility [9]. Then they can cause serious diseases because of the inherent toxicity, such as skin irritation, permanent blindness, mutation, cardiovascular disease, and mental disorders [7,10,11]. Thus, it is imperative to come up with effective strategies to separate these pollutants from wastewater before discharging.

To be accordant with the increasingly stringent environmental regulations, various advanced methods including anaerobic/aerobic biological treatment [12], electrochemical treatment [13], flocculation [14], membrane separation [15], photocatalysis [16,17], and adsorption [9] have been developed to control the dye levels of industrial effluents before discharging. Among these potential approaches, adsorption is deemed to be a simple, economical, efficient, and energy-saving measure for pollutants disposal [18,19]. In this context, considerable activated carbons have developed as adsorbents for dye separation from wastewater, but the traditional ones present high cost, poor durability and stability [20,21]. In order to figure out these issues, numerous inexpensive materials such as *Capsicum annuum* seeds [22], waste paper [4], macroalgae residue [23], municipal discarded material [24], alkali-fused fly ash/agricultural wastes [25], penicillin fermentation residue/sludge [26], wakame [10], ferrocene/toluene [27], etc., have been successfully used to prepare carbon-based adsorbents. Although these adsorbents showed attractive qualities in dye removal, they required high-temperature (≥300 °C) pyrolysis, long retention time, complicated pre-processing and structure modification. Moreover, the pyrolysis process can produce and emit harmful gas and soot into the air, and in turn cause atmospheric contamination. Therefore, the development of a novel carbon adsorbent via a truly eco-friendly and facile strategy is highly desirable.

Generally, organic dyes have complex aromatic structures and various active groups including –N=N–, –NH_2_, –SO_3_H, –COOH, –OH, etc., which provides the opportunity of interacting with adsorbents through the π-π stacking, hydrogen bonding and electrostatic attraction [3,21,28]. Consequently, the dyes can be effectively removed from wastewater. Recent reports have indicated the promising strategy of solvothermal synthesis that can carbonize small precursors into carbon-based materials (CM) with nanoscale structure at low temperature [29]. Compared with high-temperature pyrolysis, low-temperature solvothermal carbonization is more environmentally-friendly, energy-saving, low-cost and easily operated, which can generate/maintain more nitrogen/oxygen-related surface functional groups for the adsorbents without complicated treatment [30]. Citric acid, the second largest fermentation product, is an economic and renewable organic acid containing one –OH and three –COOH groups [31]. It has been an important precursor for the production of fluorescence carbon nanomaterials via solvothermal method [32]. However, researches on using it to prepare carbon adsorbents by solvothermal carbonization are extremely rare. Hence, it is of great challenge and practical values to solvothermally synthesize scalable citric acid based CM with favorable adsorption performance for dye removal.

Herein, a novel nano CM (N-CM) derived from citric acid and zinc gluconate was achieved through a one-step solvothermal method at 150 °C, which was used as an adsorbent for investigating its adsorption behaviors toward cationic MB and anionic RB19. Encouragingly, N-CM with a high specific surface area, and rich –COOH, –OH and –NH_2_ surface groups exhibited beneficial adsorption performance for both MB and RB19 at their natural pH aqueous solutions. Cationic MB gained much higher adsorption capacity due to the leading electrostatic attraction. In addition, the adsorption behaviors of MB and RB19 onto N-CM were also investigated by kinetic, isotherm and thermodynamic models. Furthermore, the obtained N-CM without surface modification showed superior selective adsorption capability toward MB in various mixed dye systems. These findings indicate that N-CM is very promising in organic dye removal from wastewater.

## 2. Materials and Methods

### 2.1. Chemicals

Methylene blue (MB, C_16_H_18_ClN_3_S) and reactive blue 19 (RB19, C_22_H_16_N_2_Na_2_O_11_S_3_) were purchased from Macklin (Shanghai, China). Zinc gluconate (C_12_H_22_O_14_Zn) and citric acid monohydrate (C_6_H_8_O_7_·H_2_O) were purchased from Aladdin (Shanghai, China). All other reagents and chemicals were purchased from commercial sources and were of analytical grade and used without further purification.

### 2.2. Preparation of N-CM

The N-CM was prepared from zinc gluconate and citric acid via a one-step microwave solvothermal method. In brief, 1.83 g zinc gluconate and 1.71 g citric acid monohydrate were dissolved in 40 mL formamide. After that, the solution was transferred into a 100 mL Teflon-lined stainless autoclave that was then sealed, followed by heating to 150 °C for 2 h at a microwave synthesizer. Subsequently, the resulting product was cooled and centrifuged at 1100× *g* for 30 min for obtaining the black N-CM. Then the N-CM solid was washed with deionized water until the supernatant was colorless. Finally, the purified N-CM was vacuum dried at 60 °C for 24 h.

### 2.3. Characterization

Fourier transform infrared spectroscopy (FT-IR) of the obtained N-CM was carried out by a VERTEX 70 spectrometer (Bruker, Karlsruhe, Germany) in a range of 400–4000 cm^−1^. The X-ray diffraction (XRD) pattern of N-CM was conducted on a D8 ADVANCE polycrystal X-ray diffractometer (Bruker, Karlsruhe, Germany) using Cu Kα radiation (Kα = 0.15405 nm). X-ray photoelectron spectroscopy (XPS) of N-CM was recorded using a Thermo Escalab 250XI spectrometer (Thermo Fisher, Massachusetts, USA) equipped with a monochromatic Al Kα X-ray source (1486.6 eV). Scanning electron microscope (SEM, JEOL JSM-7800, Tokyo, Japan), transmission electron microscope (TEM, JEOL JEM 2100F, Tokyo, Japan) and TEM-mapping were employed to determine the morphology and compositions of N-CM. Ultraviolet visible (UV-vis) absorption spectra of the samples were performed on a PerkinEkmer Lambda 45 spectrometer (PerkinEkmer, Massachusetts, USA). The Brunauer-Emmett-Teller (BET, Micromeritics ASAP2460, Georgia, USA) method was used to assess the average specific surface area, pore volume and pore size of N-CM by N_2_ adsorption-desorption. The zeta potentials of N-CM aqueous dispersions were determined by the Zetasizer Nano ZEN3600 (Malvern, London, UK). A versatile microwave synthesizer (XH-8000Plus, Beijing Xianghu Co., Ltd., Beijing, China) was utilized to prepared the N-CM.

### 2.4. Adsorption Studies

Methylene blue (MB, MW: 319.85) and reactive blue 19 (RB19, MW: 626.54) were used as adsorbates for the adsorption studies. Through adsorption experiments, the main influencing factors such as the contact time of dye with the adsorbent, initial concentrations of MB and RB19, incubation temperature and initial solution pH were investigated. Except for the temperature and pH experiments, the adsorption experiments were carried out directly with a 50 mL dye solution (MB or RB19) containing 0.5 mg/mL N-CM in a dark incubator shaker (260 rpm) at 25 ± 1 °C without pH adjustment. For exploring the kinetics of adsorption, the samples were withdrawn from the incubated dye solution at specific time intervals (0, 0.08, 0.25, 0.5, 0.75, 1, 1.5, 2, 3, 5, 8, 12, 24, 48, 72 h), followed by centrifuging at 10,000 rpm for 5 min to remove the N-CM. Then, the concentrations of residual MB and RB19 in solution were obtained via measuring the absorbance of supernatants using a UV-vis spectrophotometer at 664 nm and 626 nm, respectively. The initial pH effect on adsorption behavior varied from 2 to 11 was tuned by the SevenCompact pHmeter. All experiments were conducted in triplicates. The equilibrium adsorption capacity (*q*_e_, mg/g) of MB or RB19 onto N-CM and the % removal efficiency (*R*_e_) were calculated from the following formulas [33]:

(1) $$q_{e}=\frac{{(C}_{0}\;{-\;C}_{e})V}{w}$$

(2) $$R_{e}\;(\%)=\frac{{(C}_{0}\;{-\;C}_{e})}{C_{0}}\;\times \;100$$

where, *C*_0_, *C*_e_, *V* and *w* are the initial dye concentration (mg/L), equilibrium residual dye concentration (mg/L), solution volume (L) and adsorbent mass (g), respectively.

To explore the kinetics of adsorption of MB and RB19 onto N-CM, the experimental data were fitted with pseudo-first order, pseudo-second order, Elovich and intra-particle diffusion models. The corresponding mathematical models can be expressed as follows:

(3) $$q_{t}=q_{e}(1-e^{{-k}_{1}t})$$

(4) $$q_{t}=\frac{q_{e}^{2}k_{2}t}{{1+q}_{e}k_{2}t}$$

(5) $$q_{t}=\frac{1}{\beta }\ln(\alpha \beta t)$$

*q*_t_ = *k*_int_*t*^1/2^ + *C* (6)

where *q*_e_ and *q*_t_ (mg/g) are the amounts of MB and RB19 adsorbed at equilibrium and *t* time. *k*_1_ (min^−1^) and *k*_2_ (g/(mg·min)) belong to the kinetic constants of pseudo-first order and pseudo-second order adsorption, respectively. *α* (mg/(g·min)) is initial adsorption rate, and *β* (g/mg) associates with the relationship between attraction energy and the degree of surface coverage. *k*_int_ (mg/(g·min^1/2^)) is the intra-particle diffusion rate constant and *C* (mg/g) is a constant about the thickness of the boundary layer.

The diffusion-chemisorption model is often used to examine whether both diffusion and chemisorption control the adsorption process, which is expressed as Equation (7):

*t*^0.5^/*q*_t_ = 1/*k*_DC_ + (1/*q*_e_)*t*^0.5^ (7)

where *k*_DC_ is the diffusion-chemisorption rate constant.

The Langmuir, Freundlich, Temkin, Langmuir-Freundlich and Hill models were applied to analyze the adsorption equilibrium of MB and RB19 onto N-CM at 298 K, respectively, and they are defined as Equations (8)–(13):

(8) $$q_{e}=\frac{q_{m}k_{L}C_{e}}{{1+k}_{L}C_{e}}$$

where,

(9) $$R_{L}=\frac{1}{{1+k}_{L}C_{0}}$$

*q*_e_ = *k*_F_*C*_e_^1/*n*^_F_ (10)

(11) $$q_{e}=\frac{{\left(k_{L\text{-}F}C_{e}\right)}^{n_{L\text{-}F}}q_{m}}{{1+(k}_{L\text{-}F}C_{e}{)}^{n_{L\text{-}F}}}$$

(12) $$q_{e}=\frac{\mathrm{RT}}{B_{T}}\ln(k_{T}C_{e})$$

(13) $$q_{e}=\frac{q_{H}C_{e}^{n_{H}}}{k_{H}{+C}_{e}^{n_{H}}}$$

where *q*_e_ (mg/g) is the equilibrium adsorption capacity, *q*_m_ (mg/g) is the maximum monolayer adsorption capacity, and *C*_e_ (mg/L) is the equilibrium concentration of dye in solution. *k*_L_ (mg/L) is the Langmuir isotherm constant associated with adsorption energy, and *k*_L-F_ (mg/g) is Langmuir type constant defined by the van’t Hoff equation [34]. *R*_L_ is the separation factor, which can be defined by unfavorable adsorption if *R*_L_ > 1, favorable adsorption as 0 < *R*_L_ < 1, linear adsorption as *R*_L_ = 1, and irreversible adsorption as *R*_L_ = 0. *k*_F_ (mg/g) and *n*_F_ are the Freundlich constants signifying adsorption capacity and intensity, respectively, *B*_T_ (L/mg) is the heat of the adsorption constant, *k*_T_ (mg/L) is the Temkin constant, *q*_H_ (mg/g) is the maximum adsorption capacity of the Hill isotherm, *k*_H_ is the Hill constant, and *n*_K_ is the Hill coefficient involved with the synergistically binding interaction.

The thermodynamic parameters derived from the van’t Hoff plot were calculated to determine the thermodynamic behavior. The thermodynamic parameters are presented in Equations (14)–(16).

ΔG^Θ^ = −*RT*ln*K*_c_ (14)

(15) $$K_{c}=\frac{q_{e}}{C_{e}}$$

(16) $$\ln K_{c}=-\frac{{\Delta G}^{\Theta }}{RT}=\frac{{\Delta S}^{\Theta }}{R}-\frac{{\Delta H}^{\Theta }}{RT}$$

where ΔG^Θ^ (KJ/mol) is the Gibbs free energy change, *R* (8.314 J/(mol·K)) is the universal gas constant, *T* (K) is solution temperature, *C*_e_ (mg/L) is dye concentration at equilibrium, *q*_e_ is the equilibrium adsorption capacity at certain temperature, ΔH^Θ^ (KJ/mol) is the enthalpy change, and ΔS^Θ^ (J/(mol·K)) is the entropy change.

### 2.5. Statistical Analysis

The statistical analysis was performed utilizing IBM SPSS 20.0 software (IBM Corp., Armonk, NY, USA). Statistical data were displayed as mean ± standard deviation (SD) based on a one-way analysis of variance (ANOVA) with the Student-Newman-Keuls (SNK) test or the least significant difference (LSD) method (*p* < 0.05: significant differences).

## 3. Results

### 3.1. Structural Characterization

**Figure 1:**
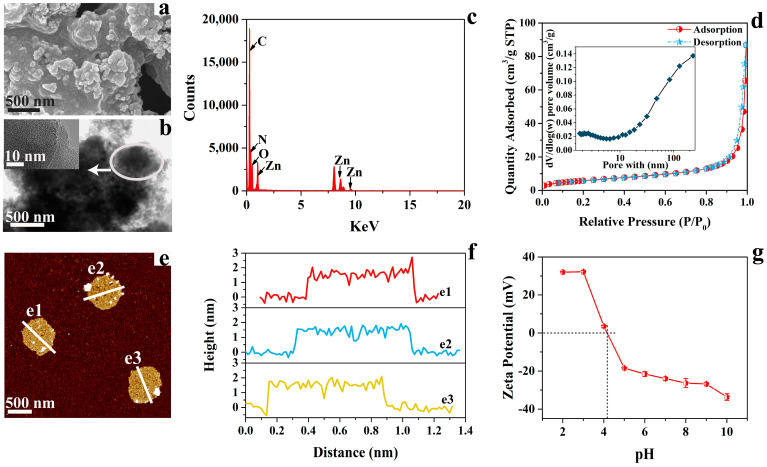
(**a**) SEM image, (**b**) TEM and HRTEM (inset) images, (**c**) TEM EDS spectrum, (**d**) nitrogen adsorption isotherms and the pore size distribution curve (insert), (**e**) AFM image and (**f**) the corresponding height distribution profile of N-CM obtained from AFM image. (**g**) The zeta potentials of N-CM in different pH environment.

Scanning electron microscope (SEM), transmission electron microscope (TEM), and atomic force microscope (AFM) images were collected to explore the morphology of N-CM. As shown by the SEM and TEM images (Figure 1a,b), N-CM is composed of a packing of ultrathin fragments that assemble into microaggregates. The high-resolution TEM (HRTEM) image (Figure 1b, inset) indicates the flake structure with disordered porous carbon of N-CM. In addition, the TEM EDS spectrum (Figure 1c) and elemental mapping images (Figure S1) validate the existence of C, N, O and Zn elements, and their uniform distribution in N-CM, where the N content is as high as 20.14%. Further, the AFM results (Figure 1e,f) show that the fragments of N-CM can assemble into quasi-disk structures with an average thickness of around 1.5 nm, which demonstrates the ultrathin nanosheet structure of N-CM. Besides, the Brunauer−Emmett−Teller (BET) measurement was performed to evaluate the specific surface area and pore size distribution of N-CM. As depicted in Figure 1d, the N_2_ isotherm for N-CM accords with the type IV isotherm, confirming the meso−macroporous feature [4,35]. The average pore size, pore volume and specific surface area were determined to be around 24.8 nm, 0.134 cm^3^/g, and 21.7 m^2^/g, respectively. The point of zero charge (*pzc*) of N-CM (Figure 1g) was calculated to be about 4.1 from the zeta potentials in various pH environments.

**Figure 2:**
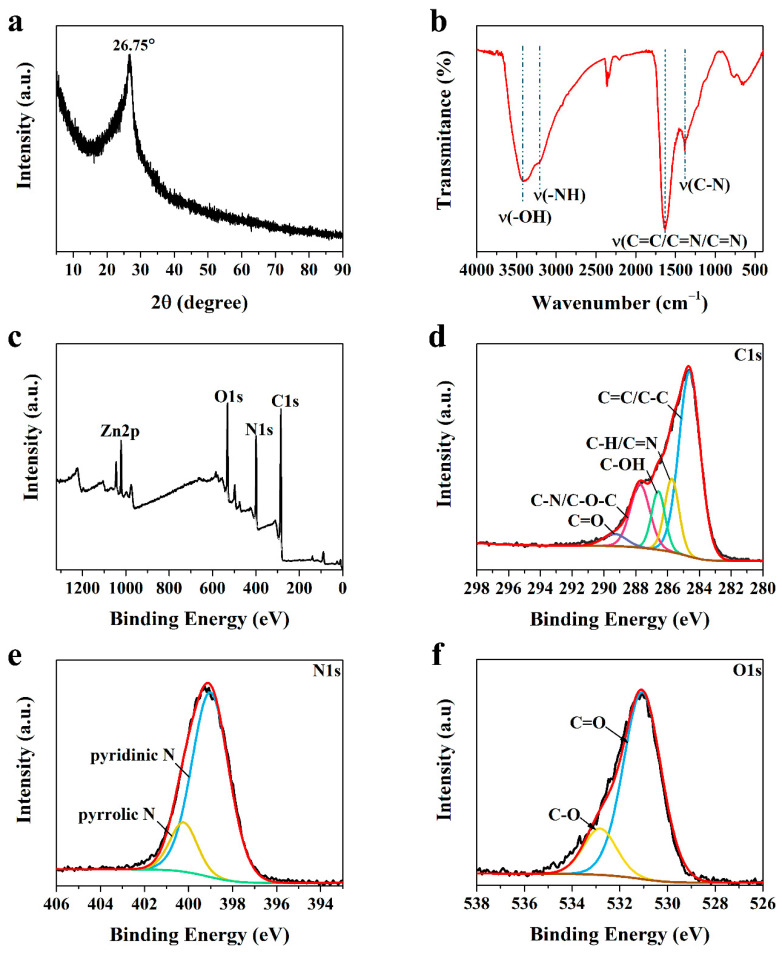
(**a**) XRD pattern, (**b**) FTIR spectrum, and (**c**) XPS survey spectra of N-CM. (**d**) C1s, (**e**) N1s, and (**f**) O1s high-resolution XPS spectra of N-CM.

The microstructure of N-CM was further characterized by X-ray diffraction (XRD) pattern. As shown in Figure 2a, a broad diffraction peak with high sharpness centered at around 26.75° was detected, which could be assigned to the amorphous phase of N-CM. The FTIR spectrum was used to uncover the surface functional groups of N-CM (Figure 2b). The broad peaks at 3409 and 3207 cm^−1^ belong to the stretching vibrations of O–H and N–H, respectively. The strong absorption band at about 1631 cm^−1^ is attributed to the vibration transitions of C=O/C=C/C=N, and the stretching vibration signal of C–N locates at 1382 cm^−1^. These results indicate that the –OH, –NH_2_, and –COOH functional groups were successfully attached to the surface of N-CM, endowing N-CM with beneficial active sites for dye adsorption.

X-ray photoelectron spectroscopy (XPS) spectra were measured to analyze the chemical compositions and surface states of N-CM. The XPS survey spectrum (Figure 2c) clearly confirms that N-CM has four elements of C, N, O and Zn, and their corresponding contents agree well with the results of the TEM EDS spectrum. The N content is calculated to be about 20.84%, further demonstrating the high N doping level in N-CM. The C1s high-resolution XPS spectrum (Figure 2d) shows five peaks at the binding energies of 284.63, 285.73, 286.58, 287.73, and 289.38 eV, belonging to C=C/C–C, C–H/C=N, C–OH, C–N/C–O–C, and C=O bonds, respectively. The N1s spectrum (Figure 2e) can be deconvoluted into two peaks at 399.03 and 400.23 eV, which are in the forms of pyridinic N and graphitic N, respectively. The O1s spectrum (Figure 2f) indicates the presences of C=O (531.08 eV) and C-O (532.83 eV) groups. These results demonstrate that N-CM possesses sp^2^/sp^3^ hybridized carbon domains, graphitic N, and abundant nitrogen/oxygen-containing functional groups, which is advantageous to the dye adsorption in aqueous environment.

### 3.2. Adsorption Study

#### 3.2.1. Adsorption Performance of N-CM toward MB and RB19

**Figure 3:**
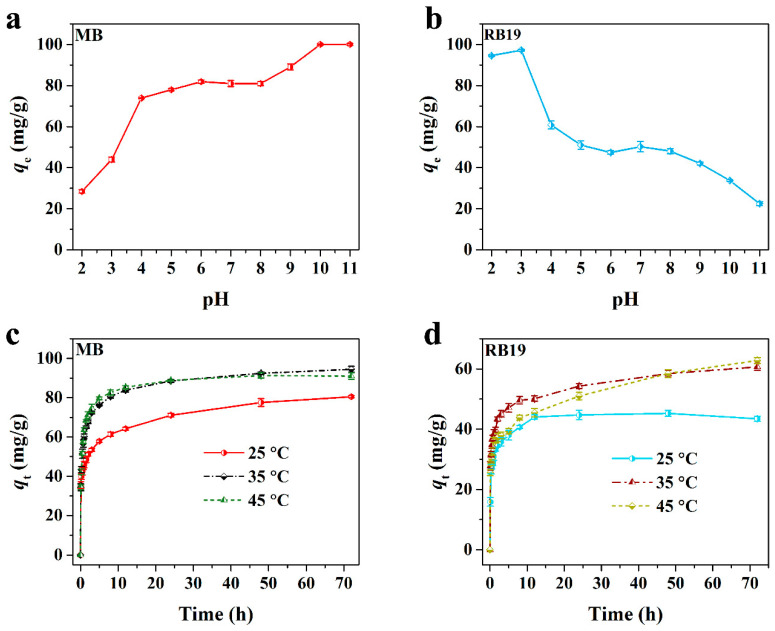
Effect of initial solution pH on (**a**) MB and (**b**) RB19 adsorption onto N-CM (at 25 °C for 72 h). Contact time effect on (**c**) MB and (**d**) RB19 adsorption onto N-CM (in natural pH aqueous solution).

In this study, the as-prepared N-CM was applied to investigate its adsorption performance toward cationic MB and anionic RB19. Generally, the initial pH of the adsorption system has a great impact on the interaction between the dye molecule and the adsorbent, and consequently affects the adsorption capability of the adsorbent [36]. Therefore, the effect of the initial pH of the dye solution on the adsorption of N-CM (0.5 g/L) toward MB and RB19 (50 mg/L) was first explored. As depicted in Figure 3a,b, the adsorption capacities of MB and RB19 showed opposite variation trends due to their different electronegativity. With the initial pH varying from 2 to 4, the MB adsorption capacity went up sharply from 28.42 to 73.92 mg/g, then slightly increased to 81.87 mg/g at pH 6.0 and remained almost unchanged at pH 6–8. An adsorption capacity of 100 mg/g was obtained when the pH shifted to 10. In contrast, nearly all the RB19 molecules were adsorbed by N-CM at pH 2–3, however, only about 50% (50 mg/g) were adsorbed in the pH range of 5–8, and continuous reduction was detected at higher pH levels due to the electrostatic repulsion between the RB19 molecules and the N-CM surface. It is demonstrated that chemisorption dominated in the adsorption processes of MB and RB19 onto N-CM. Besides, the change rule for MB and RB19 adsorption capacities in various pH environments indicates that the initial pH lower than pH*_pzc_* (4.1) was beneficial to RB19 adsorption, but higher pH was good for MB adsorption. Further, the equilibrium pH (pH_e_) values of the dye solutions after reacting with N-CM were also detected and the differences of pH_e_–pH_initial_ against pH_initial_ were compared in Figure S2a,b. The pH_e_–pH_initial_ values for both MB and RB19 were close to zero at pH_initial_ = 2, then increased to a maximum at pH_initial_ = 4, and subsequently went down to a minimum at pH_initial_ = 9/10, which also demonstrates the importance of the initial solution pH to the dye adsorption. Nevertheless, the following experiments will be performed in the natural pH dye solutions in order to better exploit the adsorption capability of N-CM toward cationic and anionic dyes, which is closer to the actual environment of wastewater treatment.

Then, the adsorption capacities of N-CM (0.5 g/L) toward MB and RB19 (50 mg/L) as a function of contact time (*t*) at 25, 35 and 45 °C were studied. It is observed in Figure 3c,d that the MB and RB19 adsorption capacities were different, while their variation trends were similar along with the increasing contact time. At the initial 5 h, the adsorption rates of N-CM toward MB and RB19 were fast, and their corresponding adsorption capacities were around 57.78 and 37.96 mg/g at 25 °C, respectively. Upon additional exposure time of N-CM to dye solutions, both MB and RB19 adsorption capacities increased gradually, and then reached a plateau. The equilibrium adsorption capacity of MB was around 80.49 mg/g, whereas that of RB19 was 45.25 mg/g, suggesting that N-CM prefers to adsorb cationic MB in the natural pH environment. The slow adsorption stage for the two dyes was probably due to the resistance from the establishment of a dye molecular layer on the surface of N-CM [10]. Moreover, when the adsorption experiments were conducted at 45 °C, the equilibrium adsorption capacities of MB and RB19 onto N-CM were determined to be around 92.41 and 62.76 mg/g, respectively, which were higher than that at 25 °C. It suggests that the increasing temperature contributes to the improvement of the N-CM adsorption capability toward MB and RB19.

**Figure 4:**
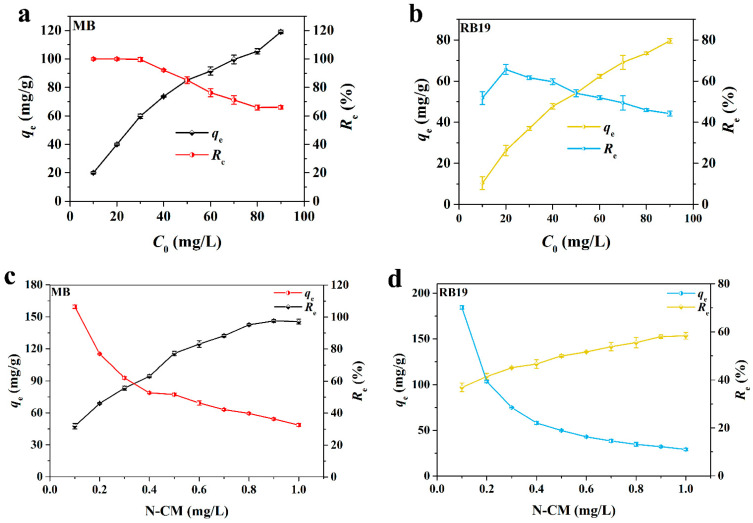
Effect of initial dye concentration on (**a**) MB and (**b**) RB19 adsorption onto N-CM. Effect of N-CM dosage on (**c**) MB and (**d**) RB19 adsorption. (in natural pH aqueous solution at 25 °C for 72 h).

The effect of the initial dye concentration on the adsorption performance of N-CM was also explored. In order to reduce the cost of pollutant removal, the adsorption temperature was set at 25 °C. As displayed in Figure 4a,b, the adsorption capacity of MB grew from 20.0 to 118.98 mg/g, and that of RB19 varied from 10.36 to 79.54 mg/g based on the initial dye concentrations ranging from 10 to 90 mg/L. In addition, the removal efficiency (*R*_e_) of MB was approximately 100% at the concentrations of 10–40 mg/L, and then reduced to 65.85% when the concentration increased to 90 mg/L; while the *R*_e_ of RB19 directly descended from 65.63% to 44.19% with the ascendance of its concentration. These findings indicate that higher initial dye concentration can not only improve the adsorption amount of dye onto the fixed dosage of adsorbent, but also intensify the competition among dye molecules on the confined active sites of the adsorbent. Besides, the increase of initial dye concentration also raises the residual dye concentration in solution, thus leading to the reduction of *R*_e_.

Since the dye adsorption also relies on the content of adsorption active sites of the adsorbent, the effect of the N-CM dosage on MB and RB19 adsorption was investigated. The experiment was implemented under an equilibrium time of 72 h and an initial dye concentration of 50 mg/mL. As illustrated in Figure 4c, the *R*_e_ of MB went up from 31.89% to 97.16% along with the N-CM amount increasing from 0.1 to 1.0 mg/mL. It is attributed to the more available active sites from N-CM and the constant MB concentration, accounting for the continuous decrease of MB adsorption capacity. Besides, Figure 4d exhibits a slow growth in the *R*_e_ of RB19 with a raise of the N-CM dosage. About 58.38% *R*_e_ of RB19 was achieved when the N-CM dosage was to 1.0 mg/mL. In addition, the RB19 adsorption capacity dropped more significantly compared to that of MB, which is likely on account of the electrostatic repulsion between RB19 molecules and the surface of N-CM.

#### 3.2.2. Adsorption Kinetics

**Figure 5:**
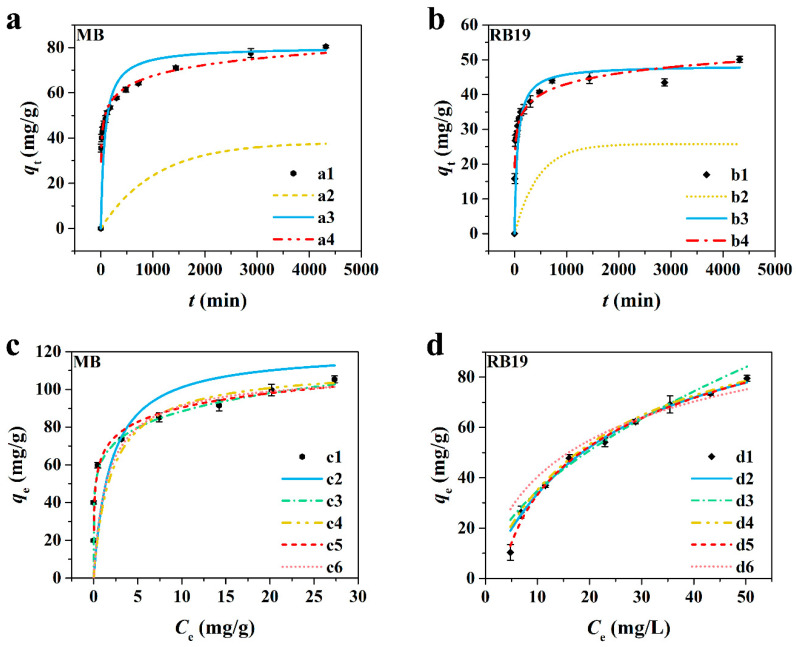
Kinetic plots for (**a**) MB and (**b**) RB19 adsorption: (a1, b1) Experimental data, (a2, b2) pseudo-first order kinetic model, (a3, b3) pseudo-second order kinetic model and (a4, b4) Elovich kinetic model. Isotherm models for (**c**) MB and (**d**) RB19 adsorption: (c1, d1) Experimental data, (c2, d2) Langmuir isothermal model, (c3, d3) Freundlich isothermal model, (c4, d4) Langmuir-Freundlich isothermal model, (c5, d5) Temkin isothermal model and (c6, d6) Hill isothermal model. (non-linear fitting; in natural pH aqueous solution at 25 °C for 72 h).

The models including pseudo-first order, pseudo-second order, and Elovich were non-linearly fitted with the experimental data to investigate the adsorption kinetics of N-CM toward MB and RB19 [37,38]. The corresponding calculated parameters are revealed in Table 1. It is observed that the pseudo-second order model exhibited a higher coefficient (R^2^) value (0.997) for MB adsorption compared with the pseudo-first order (0.902), Elovich (0.987) and IPD (0.982, 0.974) models (Figure 5a,b), demonstrating the better fit of the pseudo-second order kinetic model for MB adsorption onto N-CM [39]. Besides, the equilibrium adsorption capacity, *q*_e,cal_, of MB acquired from the pseudo-second order fitted plot is consistent with the experimental *q*_e,exp_ value (Table 1). Similarly, the RB19 kinetic adsorption conformed to the pseudo-second order model. Moreover, the fine fitting of the Elovich model for MB and RB19 adsorption with high R^2^ values (>0.95) signifies their chemisorption processes [40]. According to Figure S3g,h, the fitted plots of the intra-particle diffusion (IPD) model for MB and RB19 showed a multi-linearity deviated from the origin, indicative of the contributions of the film diffusion and intra-particle diffusion to the MB and RB19 adsorption onto N-CM [41]. The multi-linearity consisted of two stages, where the *k*_int1_ (Table 1) for the first stage was higher than *k*_int2_ for the second stage, signifying that the fast initial adsorption process was controlled by the surface diffusion [42]. To ascertain the rate-determining step for the adsorption processes of the two dyes, the Boyd model was also fitted with the experimental data. As shown in Figure S4a, the Boyd plot of MB was non-linear with deviation from the origin between *B*t vs. *t*, which could exclude the rate-determining step by intra-particle diffusion [43]. Whereas, the Boyd plot of RB19 (Figure S4b) was non-linear but passed through the origin, suggesting a significant role of intra-particle diffusion in controlling the adsorption process of RB19 onto N-CM. It indicates that the chemisorption and diffusion occurred in MB and RB19 adsorption onto N-CM. Thus, the diffusion-chemisorption model was fitted to prove the inference. As reported in Figure S4c,d and Table 1, both the plots between *t*^0.5^/*q*_t_ and *t*^0.5^ for MB and RB19 adsorption were well fitted with high R^2^ values (0.991, 0.992), and their corresponding calculated *q*_e,cal_ values were very close to the experimental *q*_e,exp_ values. This phenomenon indicates the faster surface diffusion in the MB and RB19 adsorption processes [21].

#### 3.2.3. Adsorption Isotherm

The Langmuir, Freundlich, Langmuir-Freundlich, Temkin, and Hill isotherms were applied to fit the experimental data for investigating the equilibrium adsorption of MB and RB19 onto N-CM [44,45]. The non-linearly fitted curves are displayed in Figure 5c,d, and the calculated adsorption parameters are reported in Table 2. The results reflect that the Langmuir and Langmuir-Freundlich models with higher R^2^ values (0.990 and 0.975) are more appropriate for explaining the adsorption behavior of MB onto N-CM compared with the Freundlich, Temkin and Hill models (0.944, 0.928 and 0.900). It confirms the participation of monolayer adsorption in the MB adsorption process [46]. The relevant maximum monolayer adsorption capacity was obtained as 120.77 mg/g. Besides, except for the Hill model, the equilibrium adsorption of RB19 onto N-CM was well evaluated by the Langmuir, Freundlich, Langmuir-Freundlich and Temkin isotherms (R^2^ > 0.960). Interestingly, its maximum monolayer adsorption capacity was determined to be 116.01 mg/g by the Langmuir model (R^2^ = 0.995), which is similar to that of MB. Based on the Freundlich isotherm, the 1/*n*_F_ values for both MB and RB19 adsorption were less than 1 (0 < *n*_F_ < 1), demonstrating the availability of N-CM for removing MB and RB19 from aqueous solutions. Moreover, the Langmuir-Freundlich coefficient, *n*_L-F_, and the Hill coefficient, *n*_H_, were close to 1, which suggests that the Langmuir isotherm dominates in the MB and RB19 adsorption onto N-CM [34]. Additionally, the high R^2^ values of Temkin model for MB (0.928) and RB19 (0.996) adsorption indicate the presence of chemical adsorption processes [47]. The *R*_L_ values in the range of 0–0.75 (Figure S5c,d) disclose the effective interactions between MB/RB19 molecules and N-CM [48,49]. These results indicate that the N-CM is a prospective nano-adsorbent in removing cationic and anionic dyes from wastewater. Moreover, the obtained MB and RB19 adsorption capacities were superior to some reported adsorbents, which are compared in Table S1.

#### 3.2.4. Adsorption Thermodynamics

The adsorption thermodynamic model was also used to analyze the adsorption behaviors of MB and RB19 onto N-CM. Herein, the van’t Hoff plots were fitted by ln*K*_c_ vs. 1/T and shown in Figure S6. The relevant thermodynamic parameters are reported in Table 3. In the range of test temperatures, the △G^Θ^ values were negative, substantiating the spontaneities of the MB and RB19 adsorption processes that were thermodynamically favorable. The positive △H^Θ^ values indicate that their adsorption processes were endothermic and the positive △S^Θ^ values were associated with the random growth at N-CM/dye solid solution interface [4]. In addition, the decrease in the △G^Θ^ value of RB19 under the temperature varying from 288 to 328 K testifies that increasing temperature is conducive to its adsorption onto N-CM. In the case of MB, the △G^Θ^ value gradually dropped at 288–308 K and kept nearly constant at 308–328 K, which suggests that the spontaneity of MB adsorption is not completely governed by temperature, and the 298 K will be a good choice for dye adsorption onto N-CM.

#### 3.2.5. Possible Adsorption Mechanism

**Figure 6:**
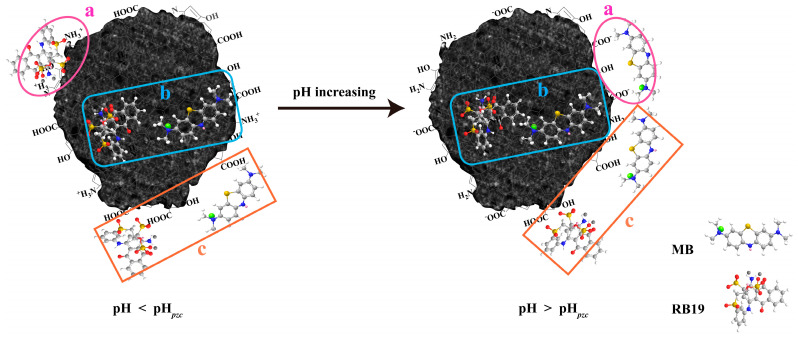
Possible adsorption mechanism of MB and RB19 onto N-CM in natural aqueous solution: (**a**) Electrostatic attraction, (**b**) π-π stacking, and (**c**) hydrogen bonding.

As-prepared N-CM with a pH*_pzc_* of 4.1 has exhibited favorable but different adsorption capacities toward cationic MB and anionic RB19 in their natural pH environment. This suggests that the adsorption behaviors of MB and RB19 onto N-CM were associated with a multi-interaction mechanism. In general, an advantageous specific surface area of 21.7 m^2^/g and wide range distribution of pore size of N-CM can be available for the physisorption of MB and RB19 via the surface diffusion and the pore pilling [21]. Further, the sp^2^/sp^3^ hybridized structure, and surface functional groups including –COOH, –OH and –NH_2_, may provide the chemisorption for MB and RB19 by the π-π stacking, electrostatic attraction and hydrogen bonding [1,11], which is presented in Figure 6. When the solution pH was lower than pH*_pzc_*, the surface of N-CM was positively charged (Figure 1g), which endowed anionic RB19 with beneficial electrostatic attraction adsorption, leading to the high adsorption capacity [7]. Whereas, it was disadvantageous to separate cationic MB due to the competition between H^+^ ions and MB molecules on N-CM [50]. On the contrary, the natural dye aqueous solution was favorable for MB adsorbing onto N-CM. As shown in Figure S7, the pH values of MB and RB19 aqueous solutions were about 6.09 and 6.86 (>pH*_pzc_*), respectively, indicating that the surface of N-CM was deprotonated and became negative in the natural pH dye solution. Therefore, negatively charged carboxyl groups of N-CM electrostatically linked to cationic MB, accounting for its significantly increasing adsorption capacity. Note that N-CM also showed decent adsorption capacity toward RB19 despite of the electrostatic repulsion between RB19 molecules and N-CM. It demonstrates that the π–π stacking and hydrogen bonding played critical roles in the adsorption of RB19 that has anthraquinone and β-sulfatoethylsulfonyl groups [51].

#### 3.2.6. Selective Adsorption

**Figure 7:**
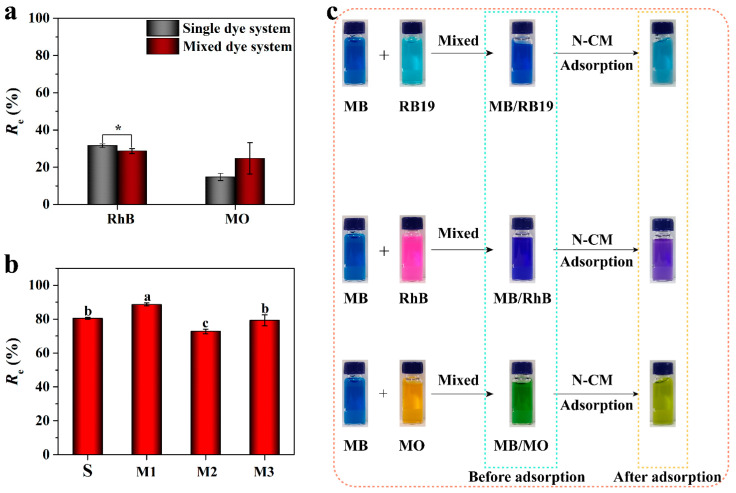
The selective adsorption of N-CM toward MB from various mixed dye systems: (**a**) The removal efficiencies of RhB (rhodamine B) and MO (methyl orange) from single and mixed dye system (one-way ANOVA with LSD test, * *p* < 0.05), vertical bars illustrate the mean ± SD (*n* = 3). (**b**) The removal efficiencies of MB from single and mixed dye system, different letters indicate the statistically significant differences (one-way ANOVA, SNK test, *p* < 0.05). S indicates the single MB dye system; M1, M2 and M3 indicate the MB/RB19, MB/RhB, and MB/MO mixed dye systems, respectively. (**c**) Photographs of the selective adsorption of MB from MB/RB19, MB/RhB, and MB/MO dye systems. (in natural pH aqueous solution at 25 °C for 72 h).

Since the adsorption capacity of MB was superior to that of RB19, it can be supposed that N-CM has selective adsorption capability toward MB. Hence, two other organic dyes (cationic rhodamine B (RhB) and anionic methyl orange (MO)) with different structures (Figure S8) were also chosen to investigate the removal performance of N-CM. As shown in Figure 7a, N-CM displayed an unsatisfying adsorption performance toward cationic RhB, with 31.61% and 28.72% *R*_e_ in the single system and mixed MB/RhB system, respectively, which were far less than that of cationic MB. It suggests that RhB adsorbed onto N-CM mainly by the hydrogen bonding, and the opposite interactions of –N(CH_3_)_2_H^+^ and –COO^-^ of RhB with N-CM presumably account for the undesirable *R*_e_ [2]. Additionally, N-CM presented poor removal efficiency (*R*_e_ ≈ 14.77%) toward anionic MO both in the individual dye solution and mixed MB/MO solution. This phenomenon is possibly attributed to the electrostatic repulsion between the anionic MO molecules and the negatively charged surface of N-CM, as well as the steric hindrance from curved conjugate structure of MO (Figure S8). The different adsorption behaviors among MB, RB19, RhB and MO suggest that the dye structure is vital to its adsorption onto the adsorbent. Figure 7b,c show the preferential adsorption of MB onto N-CM. Obviously, superior adsorption performance toward MB presented in various dye solutions. The *R*_e_ of MB increased from 80.49% in single system to 88.66% in MB/RB19 system, decreased to 72.77% in the MB/RhB system, and was nearly unchanged in the MB/MO system. These resultant *R*_e_ values confirm that a small amount of MB remained in the mixed dye solutions after treated with N-CM, causing the color of MB/RB19, MB/RhB and MB/MO mixed systems to change from dark blue, blue, and green to light blue, purple, and green, respectively (Figure 7c). The above results demonstrate the remarkable selective adsorption capability of N-CM toward MB in complex dye environments, and prove that N-CM can serve as a promising adsorbent for wastewater treatment.

## 4. Conclusions

In summary, an innovative nano-carbon adsorbent N-CM with meso-macroporous characteristic was achieved through solvothermally heating citric acid and zinc gluconate. As a carbon material, N-CM consists of nanosheet structures with sp^2^/sp^3^ hybridized domains and abundant surface groups including –COOH, –OH, and –NH_2_, which presented excellent adsorption performance toward MB and RB19. At the aqueous system without pH tuning (>pH*_pzc_*), N-CM exhibited a high adsorption capacity up to 118.98 mg/g toward cationic MB due to the dominant electrostatic attraction between MB molecules and the surface of N-CM; while an adsorption capacity of 79.54 mg/mL toward anionic RB19 because of the cooperative π-π stacking and hydrogen bonding. Nonetheless, both MB and RB19 adsorption behaviors onto N-CM were well fitted with the pseudo-second order and diffusion-chemisorption kinetic models, indicative of the multi-interaction adsorption mechanism. Moreover, the Langmuir isotherm model well described the equilibrium adsorption of N-CM toward both MB and RB19, with similar maximum monolayer adsorption capacities of 120.77 and 116.01 mg/g, respectively. Thermodynamics analysis confirms their spontaneously endothermic adsorption processes. Importantly, N-CM showed superior removal efficiencies of 72–89% toward MB in the individual MB solution, and MB/RB19, MB/RhB and MB/MO mixed solutions, substantiating the outstanding selective adsorption capability toward MB in complex dye environments. Therefore, the reported N-CM will be an effective nano-carbon adsorbent for dye contaminant removal from wastewater, especially for MB.

## Figures and Tables

**Table 1:** Parameters of kinetic models for the adsorption of MB and RB19 onto N-CM.

Kinetic Models	Parameters	Adsorbates	
		MB	RB19
	*q*_e,exp_	80.490	45.250
Pseudo-first order	*q*_e,cal_	33.369	20.437
	*k*_1_	0.00110	0.00181
	R^2^	0.717	0.816
Pseudo-second order	*q*_e,cal_	81.900	48.900
	*k*_2_	0.000112	0.000329
	R^2^	0.998	0.995
Elovich	*α*	60.529	73.183
	*β*	0.132	0.223
	R^2^	0.983	0.959
Intra-particle diffusion	*k*_int1_	1.496	1.372
	*C*_1_	34.003	21.027
	R^2^	0.984	0.979
	*k*_int2_	0.454	0.191
	*C*_2_	51.256	35.555
	R^2^	0.980	0.769
Diffusion-chemisorption	*k*_DC_	10.564	9.536
	*q*_e,cal_	89.445	51.813
	R^2^	0.994	0.993

**Table 2:** Parameters of isothermal models for the adsorption of MB and RB19 onto N-CM.

Isothermal Models	Parameters	Adsorbates	
		MB	RB19
Langmuir	*q*_m,cal_	120.773	116.009
	*k*_L_	0.515	0.0407
	R^2^	0.990	0.997
Freundlich	*k*_F_	57.489	9.816
	1/*n*_F_	0.200	0.549
	R^2^	0.944	0.979
Langmuir-Freundlich	*q*_m,cal_	112.5	119.92
	*k*_L-F_	0.480	0.041
	*n*_L-F_	0.950	0.960
	R^2^	0.975	0.972
Temkin	*B*_T_	10.987	27.760
	*k*_T_	374.398	0.331
	R^2^	0.928	0.996
Hill	*q*_H_	109.350	123.950
	*k*_H_	1.610	10.870
	*n*_H_	0.910	0.720
	R^2^	0.900	0.904

**Table 3:** Thermodynamic parameters.

Adsorbate	ΔH^Θ^ (kJ/mol)	ΔS^Θ^ (kJ mol^−1^ K^−1^)	ΔG^Θ^ (kJ/mol)				
			288 K	298 K	308 K	318 K	328 K
MB	31.169	0.122	−3.561	−5.217	−8.162	−8.034	−8.542
RB19	48.755	0.169	−1.045	−1.630	−2.780	−3.106	−7.862

## Data Availability

Data presented in this article are available at request from the corresponding author.

## Supplementary Materials

The following supporting information can be downloaded at: https://www.mdpi.com/article/10.3390/nano12111814/s1, Figure S1: The TEM EDS spectrum of N-CM: (a) C, (b) N, (c) O, and (d) Zn elements, and (e) the overlay image of these four elements. Figure S2: The pH variations between equilibrium and initiate pHs for (a) MB and (b) RB19 adsorption. Figure S3: Kinetic curves for MB and RB dyes onto N-CMs (linear fitting): (a,b) Pseudo-first order kinetic fitted plot, (c,d) Pseudo-second order kinetic fitted plot, (e,f) Elovich model, and (g,h) Intra-particle diffusion model. Figure S4: Boyd plots for (a) MB and (b) RB dye adsorption, and the linearly fitted diffusion-chemisorption model for (c) MB and (d) dye adsorption. Figure S5: Adsorption isotherm curves for MB and RB dyes onto N-CMs: (a,b) Langmuir isotherm fitted plots (linear fitting), (c,d) plots of R_L_ with initial concentration, (e,f) Freundlich isotherm fitted plots (linear fitting), and (g,h) Temkin isotherm fitted plots (linear fitting). Figure S6: The plots of ln*K*_c_ verses 1/T for adsorption of (a) MB and (b) RB dyes onto N-CMs. Figure S7: The pH of dye aqueous solution. Figure S8: The structures of MB, RB, RhB and MO dyes; Table S1: Comparison of several absorbents toward MB and RB19 dye adsorption [52,53,54,55,56,57,58,59].
