# Supplementary material for: Adsorption Behaviors of Cationic Methylene Blue and Anionic Reactive Blue 19 Dyes onto Nano-Carbon Adsorbent Carbonized from Small Precursors
Source: Nanomaterials (Basel). 2022 May 25;12(11):1814. doi: 10.3390/nano12111814 (PMC9182342; doi:10.3390/nano12111814)
Supplement: Supplementary file 1 [file nanomaterials-12-01814-s001.zip › nanomaterials-1718762-supplementary.pdf]

Supplementary Material

Adsorption behaviors of cationic methylene blue and anionic reactive blue 19 dyes onto nano-carbon adsorbent carbonized from small precursors

Caizhen Liang, Qingshan Shi, Jin Feng, Junwei Yao, Hui Huang, Xiaobao Xie,*

[1] *Institute of Microbiology, Guangdong Academy of Sciences, State Key Laboratory of Applied Microbiology Southern China, Guangdong Provincial Key Laboratory of Microbial Culture Collection and Application, Guangdong Open Laboratory of Applied Microbiology, Guangzhou 510070, PR China*

Corresponding author:

* Xiaobao Xie

Email address: xiexb@gdim.cn, Tel: +86 20 37656986

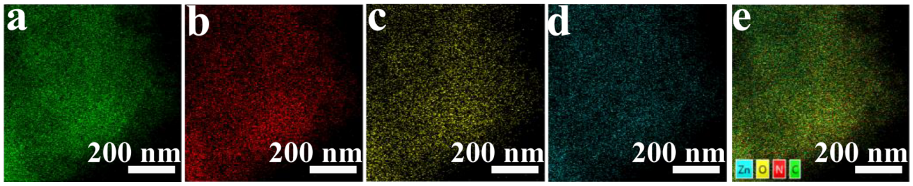

Figure S1. The TEM EDS spectrum of N-CM: (**a**) C, (**b**) N, (**c**) O, and (**d**) Zn elements, and (**e**) the overlay image of these four elements.

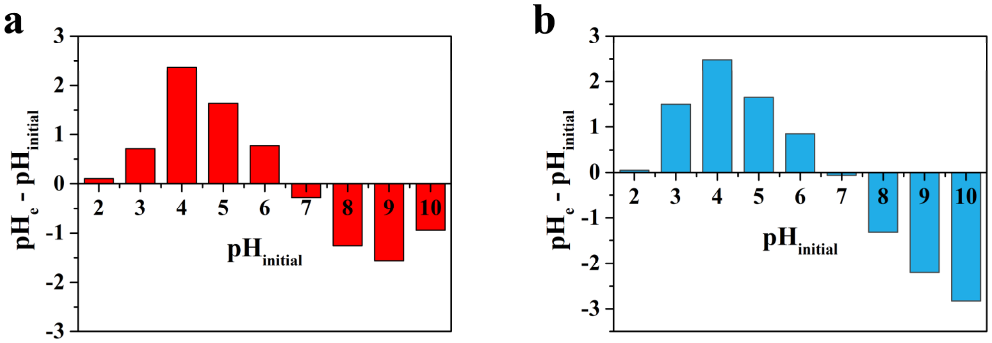

Figure S2. The pH variations between equilibrium and initiate pHs for (**a**) MB and (**b**) RB19 adsorption.

Table S1. Comparison of several absorbents toward MB and RB19 dye adsorption.

Adsorbate	Adsorbent	Synthetic Condition	Working Environment	Maximum Adsorption Capacity (mg/g)	Reference
MB	Electrolytic manganese anode slime	Hydroxylation of electrolytic manganese anode slime with EDTA-2Na	pH = 6.0	78.43 mg/g	[8]
MB	Biochar	Pyrolysis of mixed municipal discarded material at 300 °C	pH = 5 at 30 °C	7.2 mg/g	[24]
MB	Mesoporous Ni-C-N/silica aerogel	Impregnation of cationic $[Ni(trpa)]^{2+}$ complex onto silica aerogel followed by thermal treatment (200-600 °C)	pH = 4-10 at 30 °C	115 mg/g	[28]
MB	Lignocellulose hydrogel	Dissolution-regeneration of lemon peel and its microcrystalline cellulose	Aqueous solution	57.54 mg/g	[50]
MB	Cellulose-rich modified rice husk	Steam explosion and NaOH treatment	pH = 7.0	50.15 mg/g	[52]

MB	Fe_3O_4-loaded biochar	Oxygen-limited pyrolysis of $FeCl_3$-impregnated sorghum straw biomass	Aqueous solution	66.95/156.37 mg/g	[53]
MB	Mesoporous magnetic $MnFe_2O_4$@CS-SiO_2	Multi-chemical reactions containing hydrothermal synthesis	pH = 7.0 at 30 °C	179.25 mg/g	[54]
MB	Ti_3C_2 MXene@Fe_3O_4	Ti_3C_2 MXene functionalized with magnetic Fe_3O_4 via an in situ growth approach	pH = 3/11 at 55 °C	11.68 mg/g	[55]
MB	Mesoporous nitrogen-doped carbon material	Solvothermal treatment at 150 °C	Natural MB aqueous solution at 25 °C	120.77 mg/g	**This work**
RB19	Borax cross-linked Jhingan hydrogel	Borax cross-linked Jhingan hydrogel	pH = 6 at 45 °C	9.88 mg/g	[7]
RB19	Sewage sludge biochar	Pyrolysis at 450 °C	pH = 10	126.59 mg/g	[56]
RB19	Bone char	Preparation from bovine bone using CO_2 atmosphere at 451 °C	pH = 6.54	20.6 mg/g	[57]
RB19	Mesoporous activated carbon	Pyrolysis of industrial laundry sewage sludge at 750-800 °C followed by physical activation with CO_2	pH = 2.0	33.47 mg/g	[58]
RB19	Dried Aspergillus tubingensis	Isolation from filamentous fungus	pH = 2	143.0 mg/g	[59]
RB19	Nano-carbon adsorbent	Solvothermal treatment at 150 °C	natural RB19 aqueous solution at 25 °C	116.01 mg/g	**This word**

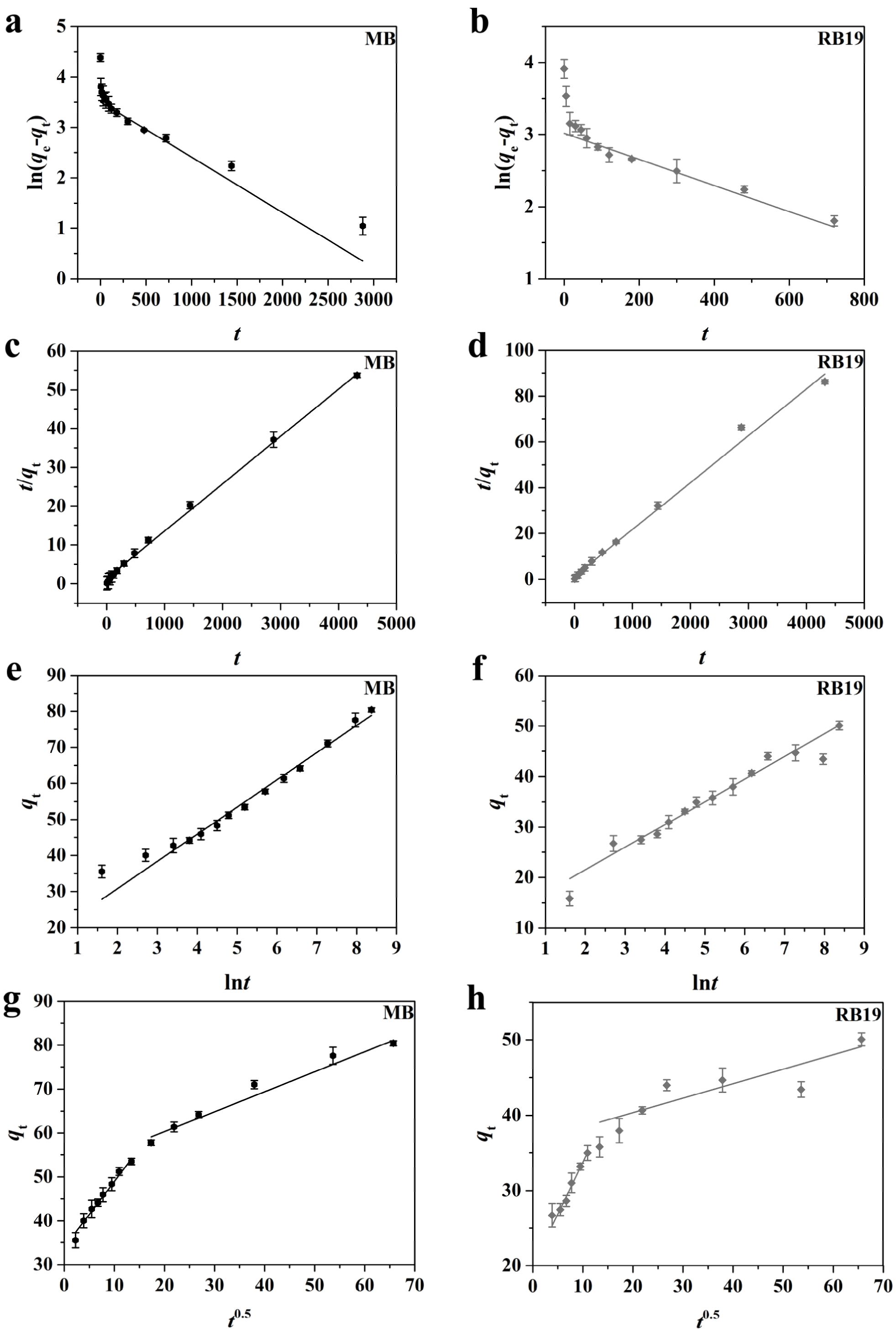

Figure S3. Kinetic curves for MB and RB dyes onto N-CMs (linear fitting): (**a**,**b**) Pseudo-first order kinetic fitted plot, (**c**,**d**) Pseudo-second order kinetic fitted plot, (**e**,**f**) Elovich model, and (**g**,**h**) Intra-particle diffusion model.

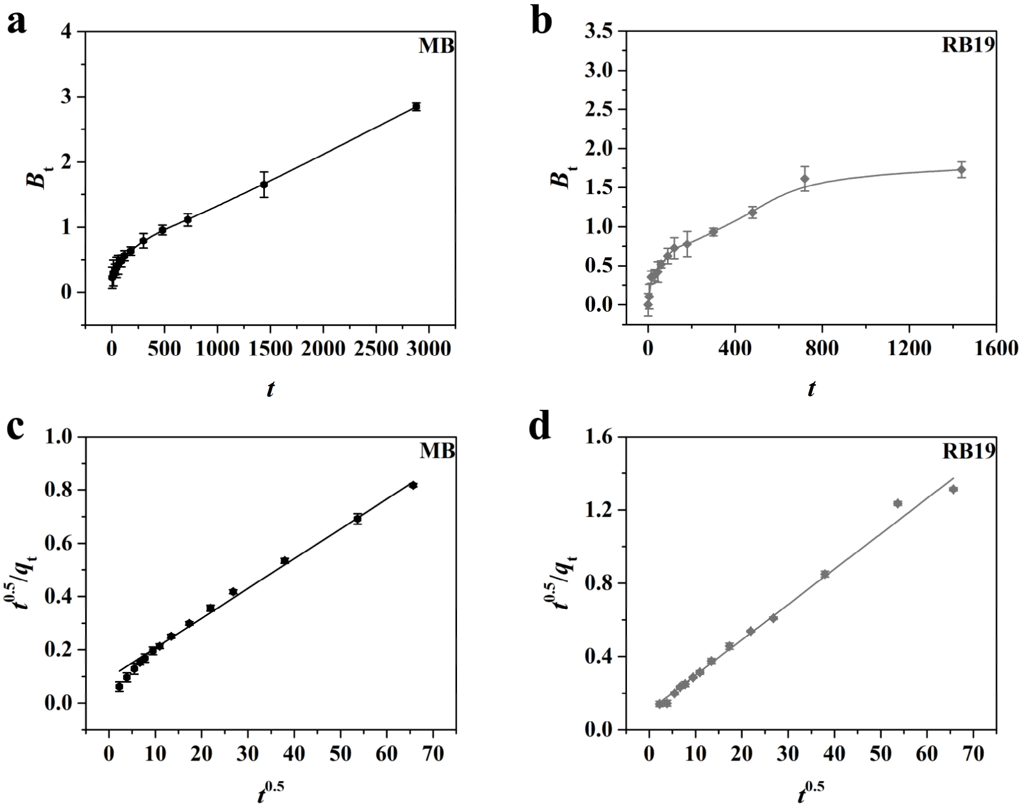

Figure S4. Boyd plots for (**a**) MB and (**b**) RB dye adsorption, and the linearly fitted diffusion-chemisorption model for (**c**) MB and (**d**) dye adsorption.

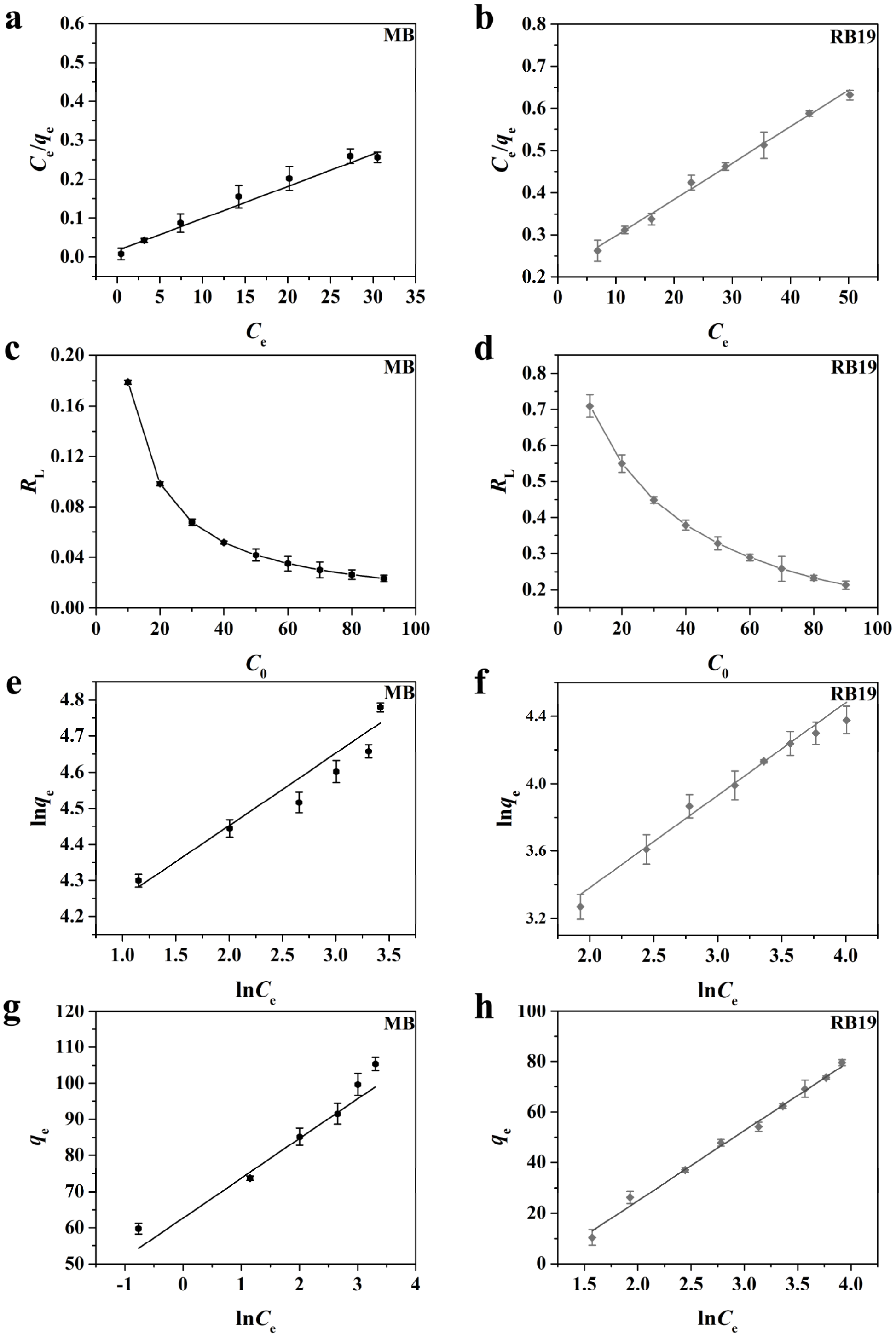

Figure S5. Adsorption isotherm curves for MB and RB dyes onto N-CMs: (**a**,**b**) Langmuir isotherm fitted plots (linear fitting), (**c**,**d**) plots of R_L with initial concentration, (**e**,**f**) Freundlich isotherm fitted plots (linear fitting), and (**g**,**h**) Temkin isotherm fitted plots (linear fitting).

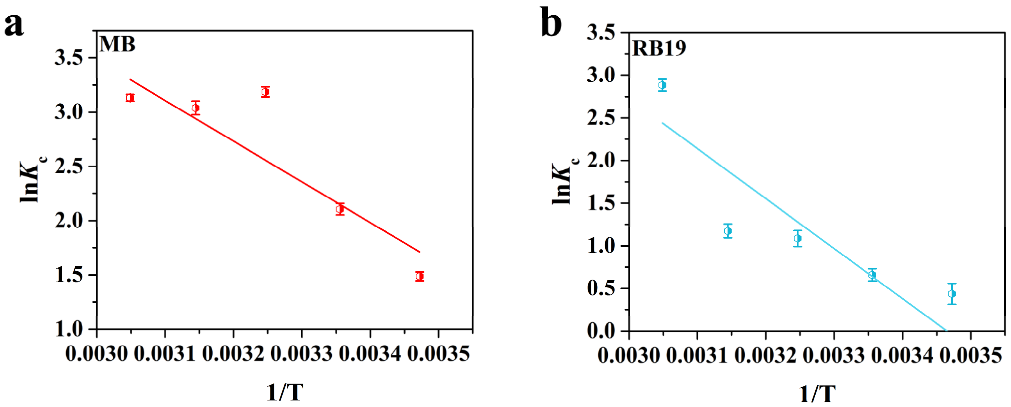

Figure S6. The plots of lnK_c verses 1/T for adsorption of (**a**) MB and (**b**) RB dyes onto N-CMs.

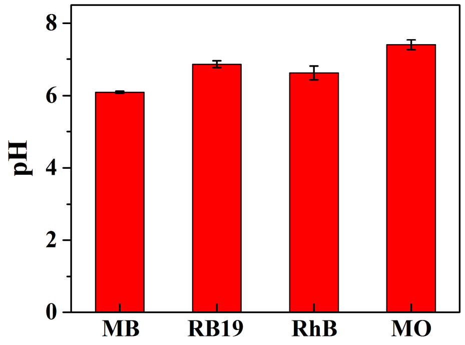

Figure S7. The pH of dye aqueous solution.

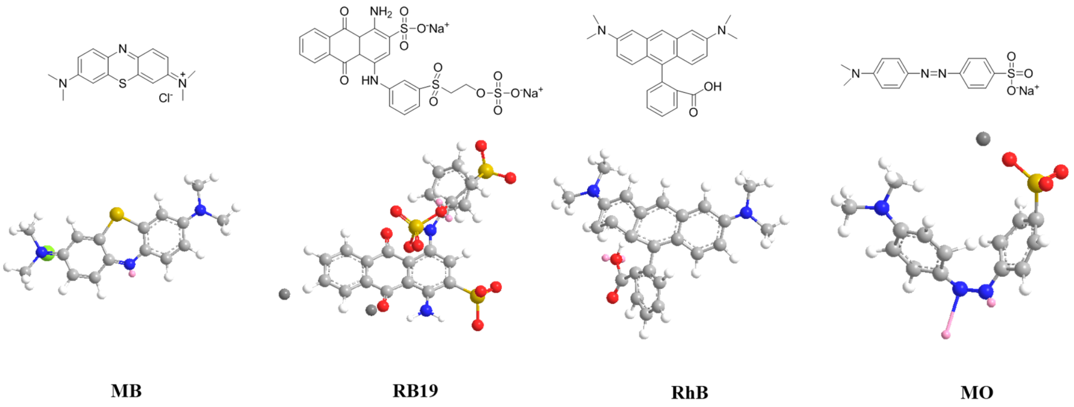

Figure S8. The structures of MB, RB, RhB and MO dyes.

References

7. Mate, C. J.; Mishra, S., Synthesis of borax cross-linked Jhingan gum hydrogel for remediation of Remazol Brilliant Blue R (RBBR) dye from water: Adsorption isotherm, kinetic, thermodynamic and biodegradation studies. *Int. J. Biol. Macromol.* **2020,** *151*, 677-690.
8. Su, P.; Wan, Q.; Yang, Y.; Shu, J.; Zhao, H.; Meng, W.; Li, B.; Chen, M.; Liu, Z.; Liu, R., Hydroxylation of electrolytic manganese anode slime with EDTA-2Na and its adsorption of methylene blue. *Sep. Purif. Technol.* **2021,** *278*, 119526.
24. Hoslett, J.; Ghazal, H.; Mohamad, N.; Jouhara, H., Removal of methylene blue from aqueous solutions by biochar prepared from the pyrolysis of mixed municipal discarded material. *Sci. Total Environ.* **2020,** *714*, 136832.
28. Dai, C.; Zhang, M.; Guo, X.; Ma, X., Mesoporous composite Ni-C-N/SA for selective adsorption of methylene blue from water. *Chem. Eng. J.* **2021,** *407*, 127181.
50. Dai, H.; Chen, Y.; Ma, L.; Zhang, Y.; Cui, B., Direct regeneration of hydrogels based on lemon peel and its isolated microcrystalline cellulose: Characterization and application for methylene blue adsorption. *Int. J. Biol. Macromol.* **2021,** *191*, 129-138.
52. You, X.; Wang, R.; Zhu, Y.; Sui, W.; Cheng, D., Comparison of adsorption properties of a cellulose-rich modified rice husk for the removal of methylene blue and aluminum(III) from their aqueous solution. *Ind. Crop. Prod.* **2021**, *170*, 113687.
53. Xie, J.; Lin, R.; Liang, Z.; Zhao, Z.; Yang, C.; Cui, F., Effect of cations on the enhanced adsorption of cationic dye in Fe3O4-loaded biochar and mechanism. *J. Environ. Chem. Eng.* **2021**, *9*, 105744.
54. Liu, Z.; Chen, G.; Hu, F.; Li, X., Synthesis of mesoporous magnetic $MnFe_2O_4$@CS-SiO_2 microsphere and its adsorption performance of Zn^{2+} and MB studies. *J. Environ. Manage.* **2020**, *263*, 110377.
55. Zhang, P.; Xiang, M.; Liu, H.; Yang, C.; Deng, S., Novel two-dimensional magnetic titanium carbide for methylene blue removal over a wide ph range: Insight into removal performance and mechanism. *ACS Appl. Mater. Interfaces* **2019**, *11*, 24027–24036.
56. Raj, A.; Yadav, A.; Rawat, A. P.; Singh, A. K.; Kumar, S.; Pandey, A. K.; Sirohi, R.; Pandey, A., Kinetic and thermodynamic investigations of sewage sludge biochar in removal of Remazol Brilliant Blue R dye from aqueous solution and evaluation of residual dyes cytotoxicity. *Environ. Technol. Inno.* **2021**, *23*, 101556.
57. Bedin, K. C.; de Azevedo, S. P.; Leandro, P. K. T.; Cazetta, A. L.; Almeida, V. C., Bone char prepared by CO_2 atmosphere: Preparation optimization and adsorption studies of Remazol Brilliant Blue R. *J. Clean. Prod.* **2017**, *161*, 288–298.
58. Silva, T. L.; Ronix, A.; Pezoti, O.; Souza, L. S.; Leandro, P. K. T.; Bedin, K. C.; Beltrame, K. K.; Cazetta, A. L.; Almeida, V. C., Mesoporous activated carbon from industrial laundry sewage sludge: Adsorption studies of reactive dye Remazol Brilliant Blue R. *Chem. Eng. J.* **2016**, *303*, 467–476.
59. Karatay, S. E.; Aksu, Z.; Özeren, İ.; Dönmez, G., Potentiality of newly isolated Aspergillus tubingensis in biosorption of textile dyes: equilibrium and kinetic modeling. *Biomass Conv. Bioref.* **2021**, *155*, 1–8.
